# Parents' Executive Functioning and Involvement in Their Child's Education: An Integrated Literature Review

**DOI:** 10.1111/josh.12612

**Published:** 2018-03-02

**Authors:** Damali M. Wilson, Deborah Gross

**Affiliations:** ^1^ School of Nursing Johns Hopkins University, 525 N. Wolfe Street Baltimore MD 21205; ^2^ Johns Hopkins University, School of Nursing, 525 N. Wolfe Street Baltimore MD 21205

**Keywords:** parental executive functioning, school achievement, parent‐school partnerships

## Abstract

**BACKGROUND:**

Parents' involvement in their children's education is integral to academic success. Several education‐based organizations have identified recommendations for how parents can best support their children's learning. However, executive functioning (EF), a high‐ordered cognitive skill set, contributes to the extent to which parents can follow through with these recommendations.

**METHOD:**

This integrative review of the literature describes how executive function can affect parents' ability to facilitate and actively participate in their child's education and provides strategies for all school staff to strengthen parent‐school partnerships when parents have limitations in EF.

**RESULTS:**

EF skills are fluid and influenced by several factors, including parental age, sleep, stress, and mood/affect. Despite possible limitations in parental EF, there are strategies school personnel can employ to strengthen partnership with parents to support their children's academic success.

**CONCLUSIONS:**

As reforms in education call for increased customization and collaboration with families, parental EF is an important consideration for school personnel. Awareness and understanding of how parents' EF affects children's learning will help schools better support parents in supporting their children's academic success.

Parents play a central role in encouraging and facilitating their children's learning and academic success from preschool through high school.^1^, ^2^, ^3^, ^4^ The Every Student Succeeds Act (ESSA), new regulations from the US Department of Education emphasizing “high‐quality, well‐rounded education” to improve student outcomes and maintain equity for all students, highlights the active role of parents.^5^ Recognizing the crucial role parents have in their children's lives, the Center for Disease Control's Healthy Schools nation‐wide initiative promotes collaboration between parents and schools to better support students' learning.^6^ Furthermore, several education‐based groups, such as the National Education Association (NEA) and the National Parent Teacher Association (NPTA), have provided recommendations for parents to support their children's learning and academic achievement (Table 1).^7^, ^8^ These recommendations are grounded in evidence about how parents and the home environment support academic success from preschool to high school, across racial, ethnic, and socioeconomic groups.^2^, ^9^, ^10^ However, these recommendations also make assumptions about parents' abilities to control, organize, and prioritize their own time and activities—cognitive skills commonly referred to as executive function.

**Table 1:** Summary of National Education Association (NEA) and National Parent Teacher Association (NPTA) Parent Recommendations for Supporting Children's Academic Success: What Is Encouraged of Parents?

Processing and communicating
Become familiar with curriculum to help prepare child Understanding of schools expectations of performance and policies (ie, homework, attendance, testing, evaluation of performance) Become knowledgeable about schools academic standing, student performance, graduation rates, and test scores Advocate for quality education (access to up‐to‐date tools and resources, safe and nurturing learning environment, small classes, ensuring educational needs are met based on child's strengths and weaknesses, interests, and learning style) Regular communication with teachers and other school personnel (via face‐to‐face time, e‐mail, phone calls, written notes)
Organizing and planning
Monitoring and guiding their child's schooling Set high expectations for their children Assist child in creating goals and plans of action to meet goals Aid in developing organizational skills (use of a planner, keep up with assignments and due dates, prioritize, plan completion based on required effort and due dates, breaking down assignments to make them more manageable) Accessing resources for tutoring, counseling, lunch programs, after‐school and extra‐curricular activities, school‐based social services, and other supplemental services
Routines and consistency
Encourage learning at home. For example, supervise and assist with homework and create a space conducive to learning (ie, eliminate distraction, provide physical space that is quiet, has appropriate lighting, and supplies). Establish daily routines. Reinforce learning at home by incorporating learned skills into everyday routines and activities Engage in activities that encourage learning (ie, visiting the museum, library, or college campuses) Volunteering in the classroom or attending meetings at the school

Executive functioning (EF) is an umbrella term referring to the complex cognitive processes that guide control and coordination of thoughts and behaviors. These skills are fluid and influenced by several factors, which may affect how parents are able to operationalize recommendations from schools. As a result, parents may appear to be ineffectual or uninvolved educational partners, despite having a great deal of interest and concern for their children's academic success. The purpose of this paper is to describe how EF can affect parents' ability to facilitate and actively participate in their child's education and provide strategies for all school staff to strengthen parent‐school partnerships when parents have EF limitations.

## METHODS

To explore parental EF and involvement in their child's education, searches were conducted in five electronic databases: CINAHL Plus, MEDLINE/Pubmed, PsycINFO, SCOPUS, and ERIC. Search criteria were set for peer reviewed research articles published from 2006 to 2016 that studied human participants and were written in English. All studies involving human subjects included an approval statement. Grey literature from well‐established and well‐known education organizations was also included. Literature pertinent to executive function, executive function in parenting, parents and child's education and academic success, and parental involvement in school, was reviewed and synthesized.

## RESULTS: LITERATURE REVIEW

Parents' EF affects their children's learning and academic achievement, in part, through its influence on parenting behaviors. Below, EF is defined and discussed in terms of how it can influence parenting and parents' abilities to support their children's academic success. Next, social, emotional, and developmental factors are identified that can diminish parents' EF. Finally, strategies are offered that can be used by schools to improve partnerships between parents and school personnel to better support children's learning and academic success even when there are parental limitations in EF.

### How EF Affects Parenting

EF describes high‐ordered cognitive processes that guide control and coordination of thoughts and behaviors mediated by the brain's prefrontal cortex.^11^, ^12^, ^13^, ^14^ EF is an umbrella term that encompasses multiple interrelated components and is most often described as having 3 domains:

- *Working memory*: Ability to retain and manipulate relevant information over brief periods of time without external aids or cues; to make planned, goal‐directed responses^11^, ^15^
- *Inhibitory control* also referred to as *response inhibition*: Ability to select a task‐appropriate, directed response over competing task‐inappropriate responses; self‐control^15^, ^16^
- *Cognitive flexibility* or *shifting*: Ability to revise actions and plans in response to situation and environment; ability to change perspectives spatially or interpersonally.^16^, ^17^

However, other tasks have also been identified as EF components including

- *Processing speed*: speed in which stimuli or information is processed to produce a response^15^, ^18^, ^19^
- *Selective attention*: filtering out unimportant information^12^, ^19^
- *Multitasking*: ability to carry out multiple tasks at once,^12^ and
- *Planning*: reasoning directed to thoughts to complete an action.^18^, ^20^

Of note, whereas domains and tasks of EF are conceptually dissociable, more practically, these domains overlap and work in various combinations to guide thoughts and produce behaviors.^11^, ^16^, ^17^ EF makes it possible for parents to play with ideas, adapt to change and challenges with speed and flexibility, take time to consider actions, reactions, and formulate plans, avoid distractions, and maintain focus.

For example, Ms. Smith sets a routine that homework and dinner preparation begin each weeknight at 5:30 pm. Her second grader is working on a book report. To prepare for the report, a schedule was set up the prior week helping her second grader read sections of the book nightly before bed. Her seventh grader, who struggles with math, needs to complete a math assignment. Recognizing the math concepts is more advanced than she anticipated, she refers her child to a list of supplementary math resources provided by the student's teacher in the course syllabus. Ms. Smith does this while preparing the family meal and maintaining a calm demeanor.

In the above example, Ms. Smith uses a number of EF skills: holding her goals in mind (working memory), focusing on what is relevant and appropriate to meeting these goals (inhibitory control/response inhibition), and resisting internal and external distractions (cognitive flexibility or shifting). Here, Ms. Smith is planning, multitasking, giving selective attention and simultaneously engaging her working memory, response inhibition, and cognitive flexibility.

Commonly acknowledged parenting tasks include: (1) giving a child attention, (2) generating appropriate (and inhibiting inappropriate) responses to a child's behaviors, (3) flexibly switching attention as situations and environments change, (4) prioritizing tasks, (5) retaining information about a child, (6) manipulating an environment to meet the child's needs, (7) evaluating outcomes and adjusting behaviors as needed, and (8) problem solving.^21^, ^22^, ^23^ All of these tasks require cognitive input and belong to domains of executive function. Table 2 provides general examples of tasks associated with the 3 major domains of EF as well as examples of parent‐specific tasks in facilitating and participating in their children's education.

**Table 2:** Examples of Parenting Tasks Requiring Executive Function

	Executive Function	Example	Application of EF to Parental Support in Child's Education
Three major domains of EF	Working memory	Hold auditory and visual information, access when needed, and maintain as new information arrives and should be incorporated Used to do an activity and pay attention	Remembering homework assignment due dates and instructions Devising a plan to complete assignments Ability to apply and expound on previous lessons and assignments
	Inhibitory control/response inhibition	Avoid distractions Being thoughtful in response to an event or situation opposed to reflexive, first reaction	Not abandoning a school task in favor of a more exciting or pleasurable activity Avoiding distraction from other situational or life events to support child's learning Stopping oneself from reacting negatively if child is struggling
	Cognitive flexibility/shifting	Shift attention, tasks, or roles easily and quickly Adapt to change	Assisting multiple children in differing grade levels with assignments at once Recognizing that homework plan may need to change based on child's mood or abilities, and adjusting accordingly

EF, executive functioning.

It is well established that parental EF plays a significant role in parenting behaviors and practices. In 2005, Azar and Weinzierl published a cognitive behavioral model (CBT) discussing capacities crucial to parenting, one being EF.^22^ Within the context of changing developmental needs and capacities of children, the CBT model posits that parents are continuously faced with situations that engage EF. Aspects of and variations in EF therefore contribute to parenting, such as skills to meet child's physical and emotional needs, ensure safety from physical and psychological harm, provide warmth and nurturance, interact sensitively, and apply positive and consistent discipline. 22 EF also affects the ability to parent calmly despite environmental conditions or child responses, when the parent is strained, in the setting of challenges, and within changing needs of the parent and child (eg, managing a tantrum in a grocery store, parenting despite stressful family relationships).^22^ Parental EF therefore contributes to the quality of parenting practices and parent‐child interaction.

Gonzalez^24^ and Crandall et al^25^ propose models that account for the intergenerational impact of parental EF where delays and deficits in EF impact parenting and more distally, the socioemotional, behavioral, cognitive, and developmental outcomes of their children. For example, these parental delays and deficits in EF can impact child outcomes via limited maternal sensitivity, timely, and appropriate responses to child's cues and poor attachment quality.^24^, ^25^ These have implications for social and cognitive development; decreased vocalization and scaffolding with a child, and in extreme cases, the sequelae of child maltreatment, which can persist into adulthood including behavioral problems and neurocognitive delays in attention, memory, inhibition, problem solving, and planning.^25^ Consistent with these models, parental EF has been linked to parental negativity and harshness, reactivity to child emotions and behaviors, facilitating attention, physical stimulation, intrusiveness, and sensitivity, where poorer EF has been associated with the more negative behaviors and higher EF associated with more positive behaviors and interactions.^26^, ^27^, ^28^, ^29^, ^30^, ^31^ Johnston et al found that EF deficits impact parenting control and responsiveness that can manifest as inconsistent and overreactive discipline, challenges in prioritizing and organizing parenting tasks, lack of warmth or sensitivity, poor guidance or monitoring, and challenges in problem solving.^32^ All these sequelae of parental EF can affect the ability of parents to meet the important expectations schools hold for parents listed in Table 1. Given what is known about the importance of EF in coordination and control of thoughts and behaviors, and the impact parental EF has on parenting behaviors and interactions, it becomes clear that EF is also a contributor to the parent's ability to facilitate and actively participate in their child's education or the quality of that ability.

It is important to note that parental EF is fluid. That is, EF is a cognitive capacity subject to influence and fluctuations by various developmental and psychosocial factors.^25^ These factors can help explain challenges, limitations, and variability in parental EF that might impact a parent's ability to meet school involvement expectations, such as those listed in Table 1.

### Factors Influencing Parents' EF?

#### *Parent age*

Research literature has established that structural and functional changes in the brain occur across childhood and during puberty and adolescence, specifically in the brain's prefrontal cortex, which mediates EF.^12^, ^13^, ^14^, ^19^ Accordingly, there is consensus that maturation and refinement of EF skills takes place throughout adolescence and into early adulthood.^11^, ^13^, ^14^, ^26^ Early development is the acquisition of abilities, while maturity through adolescence and emerging adulthood is the sophistication of these abilities.^15^ On the other end of the age continuum, changes in brain structures such as decreased size and integrity, and declines in EF abilities (eg, processing speed, working memory, inhibitory control, and cognitive control) are considered normal with aging.^33^, ^34^ Similar to development in adolescence, tasks of EF display heterogeneous patterns in aging.^35^ Whereas there is much variability across individuals, parent age may be an important factor to consider how EF skills are employed among adolescent and young adult parents, older parents or grandparents who serve as primary caretakers. Widening trends in the age of primary caregivers for children enrolled in school have implications for parents' EF skills relevant to supporting their children's educational success.

#### *Sleep*

Approximately, 25% of adults report insufficient sleep or rest.^36^ Sleep deprivation compromises alertness, attention, and vigilance,^37^, ^38^ reduces effective communication, and impairs decision making in the setting of managing the unexpected, distractions, and revising plans.^39^ All these play a role in parenting and parental involvement with education in particular. The results of a meta‐analysis showed the deleterious effect of poor sleep on working memory.^40^ The significance of sleep deprivation on response inhibition is established as well as effects on cognitive flexibility.^41^, ^42^ The interaction between sleep and EF may be an important consideration for parents who work long hours, work at night, or hold multiple jobs. Parents' work schedules can greatly impact their physical and psychological availability to engage in their child's education as well as the EF skills required to support their child's academic success.

#### *Stress*

Chronic or repeated exposure to stress has enduring effects on the brain.^43^ It is therefore no surprise that various forms of stress have been linked to impaired EF in adults; for instance, acute stress on working memory,^44^ as well as perceived stress on attention, working memory, and processing speed.^45^, ^46^ Although higher stress levels are associated with poorer EF, the interaction between these 2 variables is complex. For example, fluctuations in naturally occurring daily stress (eg, having an argument with someone, something happening to a close friend, a situation around personal health) have been associated with fluctuations in EF. 47 Additionally, stressors known to cause structural changes to the prefrontal cortex and measurable impairments in EF have proven reversible after removal of the stressor.^48^

There are individual differences in how people perceive, experience, and respond to stress even under similar conditions (similar stressor, resources, history, environments). Stress is a contextual‐specific individual response to a stimulus, making it subject to great variability.^49^ The influence of stress on EF is an important consideration as stress is a normal part of life as an adult as well as functioning in the role of parent. The experience of stress may explain how a parent who was previously highly engaged becomes less attentive to their child's work, less involved in school‐related activities, or may have difficulty keeping up with forms and meeting deadlines.

#### *Mood and affect*

Recent evidence confirms that emotions and mood impact EF. Anger rumination, a tendency to think repeatedly about the implications, causes, and meanings of an angry mood, has been associated with worse performance in shifting and response inhibition.^50^ EF impairments have been associated with anxiety,^51^ more specifically attentional control, one's ability to engage and disengage attention to aspects of the environment^52^ and shifting.^51^, ^53^ Further, anxiety/worry has been shown to affect working memory.^54^ There is extensive evidence of difficulties in EF secondary to depression.^55^, ^56^, ^57^, ^58^ On the other hand, positive emotions have been associated with more optimal EF. For example, happiness has been linked to some improvement in working memory,^59^ and positive affect can improve task‐switching abilities.^60^ These associations between mood and affect and EF may be an important consideration for determining ideal times to engage parents and also identifying situations that may require deference or de‐escalation; for example, in situations where a parent is visibly angry or anxious.

## DISCUSSION

In December 2015, ESSA was signed by President Obama, with final regulations announced by the Department of Education in November 2016. This legislation is a shift from the Elementary and Secondary Education Act of 1965, and its subsequent amendment, the No Child Left Behind Act of 2001. ESSA takes a more holistic approach, requests flexibility in specific requirements, and gives more autonomy to states and districts to prepare their students for success in college and careers.^5^, ^61^ In this age of education reform, the growing interest in customized education for students should be matched with tailored expectations and recommendations for parents to better facilitate collaboration and partnerships.^62^ Additionally, school leaders have been called on to develop more expansive and engagement focused connections with students' families as a means to improving student achievement.^63^

Multiple factors contribute to parents' capacities, skills, and performance in supporting their child's readiness to learn and academic success. EF is one such contributor. EF is a learned and cultivated skill set but it is also fluid. EF can fluctuate when parents are tired, stressed, or depressed. This is also an important consideration in challenging assumptions and biases related to how parents engage and invest in their children's education that may vary from the ways school personnel typically expect. School personnel understanding how EF can influence parents' abilities to be actively engaged in their children's education is essential for supporting parents and helping parents support their children. Last, education policy should continue to ensure all school professionals are trained to approach and interact with parents and families encompassing a holistic view of the parent and expand to include factors such as parental EF.

## IMPLICATIONS FOR SCHOOL HEALTH

There is robust evidence that practice and training using a variety of modalities improve EF skills in children.^64^, ^65^, ^66^, ^67^, ^68^ Among adults, however, the evidence is less compelling. There is some evidence that training can improve skills, but improvements are effortful, appear to be short term, and success in one skill does not readily generalize to success in other skills or situations.^25^, ^69^, ^70^, ^71^ Therefore, the following recommendations are not designed to change parental EF but offer strategies for schools to partner with parents who have limitations in their EF. These recommendations are grounded in the science of EFs and skill development of EF among young people adapted to the recommendations in Table 1 for parents.

1. Identify and create specific, meaningful educational goals and plans with parents; start with simple and achievable goals before moving on to more complex or longer term goals.^72^, ^73^ There is an ability to choose voluntarily to attend to or ignore stimuli based on goals or intention.^17^

*Example*: Start conversations allowing parents to share their expectations and goals for their child's education for the school year. Also share your goals and plans. Discuss how each party contributes to attainment of those education goals. Establish timelines together. Share support services that can be helpful in having educational goals met. Acknowledge stated challenges and limitations shared by parents. Focusing on the positive helps establish rapport and helps solidify shared goals. Contact parents outside of a problem or concern, like when a student does a nice job on an assignment or is helpful to a classmate.

- Have a standard motion or sound that signals a call for attention; for example, in a group setting. There is a natural tendency for a salient stimulus, for instance, a visual motion or a loud noise, to command attention when it appears.^17^

*Example*: Routinely start large meetings with a distinct sound and/or a unique hand signal to serve as a call for attention.

- Use concise, focused messages whenever possible.

It is more difficult to hold several ideas in mind as compared to only 2 or 3. Also clear, brief messaging helps keep mental workspace from becoming too cluttered.^17^

*Example*: Use concise statements and phrases to reinforce messages in flyers, posters, or e‐mails. Focus on what is most important at the time or in the near future versus a long narrative where key information can be lost and clouded by less important information. For example: “Permission slips due Friday for field trip,” “Math tutoring for 3rd graders every Tuesday,” “Registration for fall classes ends May 31st.”

- Make actionable items simple and stepwise; clustering‐related actions together.^72^

When instructed on multiple tasks, individuals with worse EFs would likely fail to switch between tasks as they should or perform less optimally on tasks. It is also harder to clear an irrelevant task from their mental workspace.^17^

*Example*: If there is a process for submitting school health forms online, provide the step‐by‐step instructions in a list form. Use clear, simple language. Provide pictures or screenshots. Avoid including other information not pertinent to the task of submitting the health forms.

- Concrete and consistent reminders. Use cues, visual and/or auditory, to reinforce messages.^72^ Cues and reminders help bring thoughts and actions into the consciousness over time. People are quicker to notice, and respond to, stimuli already being held in mind. By concentrating information you increase the likelihood that that information will be held and will guide behavior.^17^

*Example*: Use flyers, posters, e‐mails, phone trees, automated calls, text messaging, mailers, bulletin boards, school website, mailed save‐the‐dates, and social media to deliver consistent, concise messaging. Perhaps provide a school calendar in the beginning of the year with all meetings, major events, and important deadlines but also provide quarterly, monthly, or weekly reminders as appropriate.

- Be flexible and consider different perspectives.^72^ In terms of EF, people generally like everything to stay the same or everything to change.^17^ If a particular message or task does not seem to be translating well to parents consider the following: “What might we do differently?” “What other approach may be appropriate?” “How can we present the material differently?” “Word the information differently, so that parents can understand and engage?” Use of technology may be helpful as well.

*Example*: If there is poor attendance at parent‐teacher conferences, consider an alternative day or time, perhaps offer telephone or Skype appointments. Color code important dates based on priority on flyers and mailers to increase attention to important details.

- Help establish and maintain routines.

It is not as demanding for adults to keep doing something consistently, even if it is counterintuitive or counter to their initial inclination as over time it becomes almost automatic.^17^ What is most challenging is switching back and forth between ways of thinking about or acting in response to a stimulus. The way something becomes second nature or automatic is through repeated practice.^17^

*Example*: Standardize due dates, for example, book reports are due the first Friday of every month, Show and Tell is every Monday. Have a homework folder that parents must sign off on every week. Share the class schedule with parents. Provide a checklist for assignments and activities.

- Be supportive. Create a school community. Recognize that all parents, regardless of age, income, employment, education, or cultural background, want to be involved in their children's education and want their children to do well in school. People perform better and show better EF if they feel they are in a supportive community, one that is inviting, consistent and dependable.^17^ A previous study found that parental perceptions and experiences with the school impact involvement where parents who reported more contact from the school and higher levels of school outreach were more involved with children's schooling^74^

*Example*: Listen. Show a willingness and interest in learning about the student from their parents. Include students in parent conferences. Visit parents directly at their homes or other safe, comfortable locations. Invite parents into the classroom. Initiate school‐wide programs such as family math night or exercise night. Establish an open‐door policy between parents and school personnel. Create an anonymous suggestion or comment box. Embrace a philosophy of partnership. Create a parent buddy system whereby 2 parents are matched with the ability to connect with one another for support, encouragement, or verification of schools' processes.^73^
